# Microbial Community Field Surveys Reveal Abundant *Pseudomonas* Population in Sorghum Rhizosphere Composed of Many Closely Related Phylotypes

**DOI:** 10.3389/fmicb.2021.598180

**Published:** 2021-03-09

**Authors:** Dawn Chiniquy, Elle M. Barnes, Jinglie Zhou, Kyle Hartman, Xiaohui Li, Amy Sheflin, Allyn Pella, Ellen Marsh, Jessica Prenni, Adam M. Deutschbauer, Daniel P. Schachtman, Susannah G. Tringe

**Affiliations:** ^1^Department of Energy, Joint Genome Institute, Berkeley, CA, United States; ^2^Lawrence Berkeley National Laboratory, Environmental Genomics and Systems Biology Division, Department of Energy, Berkeley, CA, United States; ^3^Department of Horticulture and Landscape Architecture, Colorado State University, Fort Collins, CO, United States; ^4^Department of Agronomy and Horticulture and Center for Plant Science Innovation, University of Nebraska-Lincoln, Lincoln, NE, United States

**Keywords:** microbial profiling, rhizosphere microbial communities, high-throughput 16S rRNA gene sequencing, microbiome, *Pseudomonas*, sorghum

## Abstract

While the root-associated microbiome is typically less diverse than the surrounding soil due to both plant selection and microbial competition for plant derived resources, it typically retains considerable complexity, harboring many hundreds of distinct bacterial species. Here, we report a time-dependent deviation from this trend in the rhizospheres of field grown sorghum. In this study, 16S rRNA amplicon sequencing was used to determine the impact of nitrogen fertilization on the development of the root-associated microbiomes of 10 sorghum genotypes grown in eastern Nebraska. We observed that early rhizosphere samples exhibit a significant reduction in overall diversity due to a high abundance of the bacterial genus *Pseudomonas* that occurred independent of host genotype in both high and low nitrogen fields and was not observed in the surrounding soil or associated root endosphere samples. When clustered at 97% identity, nearly all the *Pseudomonas* reads in this dataset were assigned to a single operational taxonomic unit (OTU); however, exact sequence variant (ESV)-level resolution demonstrated that this population comprised a large number of distinct *Pseudomonas* lineages. Furthermore, single-molecule long-read sequencing enabled high-resolution taxonomic profiling revealing further heterogeneity in the *Pseudomonas* lineages that was further confirmed using shotgun metagenomic sequencing. Finally, field soil enriched with specific carbon compounds recapitulated the increase in *Pseudomonas*, suggesting a possible connection between the enrichment of these *Pseudomonas* species and a plant-driven exudate profile.

## Background

The plant microbiome is a dynamic landscape, shifting both over plant development and under environmental change (Edwards et al., 2018; Xu et al., 2018; Zhalnina et al., 2018). Typically, the community of microbes living in close proximity to the root, commonly referred to as the rhizosphere, exhibits a level of diversity that is lower than that of the surrounding bulk soil community, but higher than that of the root endosphere, which is more tightly regulated by the host plant (Hacquard et al., 2015). As a result, dominance of a single bacterial lineage in the rhizosphere is rarely seen, except in the context of clonal expansion of plant pathogens common with disease outbreaks (Kolmer, 2005; Cai et al., 2011; Butler et al., 2013; Yoshida et al., 2013).

However, several recent microbiome surveys in diverse plant hosts detail examples of plant-associated communities dominated by the bacterial genus *Pseudomonas*. Large scale surveys using 16S rRNA gene amplicon sequencing of the maize rhizosphere collected from field grown plants across two growing seasons in New York revealed that just three *Pseudomonas* OTUs accounted for ∼44% of the maize rhizosphere amplicon reads beginning after week 8 in one of these seasons (Walters et al., 2018). Interestingly, this enrichment was not seen in the surrounding soil, indicating that plant selection, possibly combined with the opportunistic nature of these microbes, was driving their increased abundance. In wild populations of *Arabidopsis thaliana* collected in Southwestern Germany, a single *Pseudomonas* OTU accounted for ∼50% of amplicon sequences in the phyllosphere (Karasov et al., 2018). Isolate sequencing revealed this highly dominant OTU as *P. viridiflava* from the *P. syringae* complex, a putatively pathogenic species in *Arabidopsis* (Karasov et al., 2018). Additional *Pseudomonas* dominated rhizosphere surveys have been documented in maize and wheat (Ofek et al., 2014). Collectively, these studies present instances of *Pseudomonas* dominance within different plant compartments, soil types, plant hosts, and geographic locations, and highlight the need for improved understanding of the community dynamics and plant microbe interactions that produce them.

*Pseudomonas* are gram-negative, oxidase positive, and non-spore forming bacteria that are known for inhabiting a diversity of environments (Garrity et al., 2005). Additionally, this genus’ association with plant microbiomes is well documented and it possesses many genomic traits that support plant colonization. *Pseudomonas* spp. are opportunistic with multiple efflux pumps that can expel a broad range of antibiotics (Raaijmakers et al., 2002; Haas and Keel, 2003; Igbinosa et al., 2012), making them excellent at microbial warfare and strong competitors for the limited plant resources. They are also motile, having one or more flagella, an important feature for plant colonization (Lugtenberg et al., 2001; Cole et al., 2017). The genus *Pseudomonas* contains many species considered to be plant growth promoting rhizobacteria (PGPR) (Lugtenberg and Kamilova, 2009). There are also *Pseudomonas* spp. that are pathogenic to plants, such as *P. syringae* pv. Tomato DC3000, which infects the plant phyllosphere (Worley et al., 2013). Other *Pseudomonas* have a plant species-specific effect in priming the host’s immune system through induced systemic response (ISR) (Berendsen et al., 2015). Finally, the *Pseudomonas* genus is one of the largest and most diverse in the bacterial kingdom (Gomila et al., 2015), which has impeded efforts to accurately dissect and taxonomically classify phenomena involving *Pseudomonas* using traditional microbiome analysis platforms.

Traditional methods of analyzing microbiome composition often rely on clustering sequences into Operational Taxonomic Units (OTUs) at a fixed similarity threshold, typically 97%. The usefulness of this approach has been recently called into question (Koeppel et al., 2008; Shapiro and Polz, 2014; Tikhonov, 2017), as it may not be sufficient to identify sub-genus and subspecies trends that are important for understanding functional and ecological relationships. This may be particularly true for large and diverse lineages, such as *Pseudomonas*, as OTUs identified as *Pseudomonas* may comprise multiple strains or species that share a core genome, but which differ substantially in their accessory genomes (Tettelin et al., 2005). While in the past clustering at 97% identity was done in part to reduce influence of artifactual clusters introduced through sequencing error (Koeppel and Wu, 2013), recent advances in sequencing error-correction algorithms now permit the taxonomic investigation of sequenced amplicons at finer scale resolution without this issue. These newer tools perform analyses using Exact Sequence Variants (ESV) and can provide higher resolution insights into how microbial communities change over time or across different ecological niches (Callahan et al., 2017; García-García et al., 2019). Additionally, long-read technologies that allow for full-length 16S rRNA sequencing can aid efforts to catalog and classify reads (Singer et al., 2016), and may be particularly helpful for assigning taxonomy to organisms belonging to large and highly similar phylogenetic groups, in which individual subspecies can share 100% sequence identity over small regions of the 16S marker gene, preventing even ESV clustering methods from assigning them to distinct units. Importantly, it has been shown that approaches that allow higher-resolution taxonomic classification can reveal previously overlooked patterns of ecological distribution across environments or experimental treatments (García-García et al., 2019).

Here, we report the dominance of a single bacterial OTU from the genus *Pseudomonas* in the rhizosphere microbiome of *Sorghum bicolor*, a plant species for which *Pseudomonas* enrichment has not previously been reported. Using 16S rRNA gene amplicon sequencing, we profiled the microbial communities of root, rhizosphere, and bulk soil samples collected at 7 and 15 weeks post-planting from 10 sorghum genotypes grown under high and low nitrogen (N) field conditions in eastern Nebraska. We found a predominance of *Pseudomonas* within rhizosphere samples collected at 7 weeks across all plant genotypes and present in both low and high nitrogen fields. We subsequently explored the intra-OTU diversity using a combination of ESV-based clustering methods and long-read sequencing technologies to gain insights into the phylogenetic structure of this group of *Pseudomonas* and their potential relationship with the sorghum host. We demonstrate that the observed *Pseudomonas* bloom comprises a taxonomically broad group of soil-derived *Pseudomonas* lineages, rather than being dominated by a specific *Pseudomonas* lineage. Additionally, we show that *Pseudomonas* blooms can be observed in enrichment experiments on specific plant-derived carbon sources.

## Results and Discussion

**FIGURE 1:**
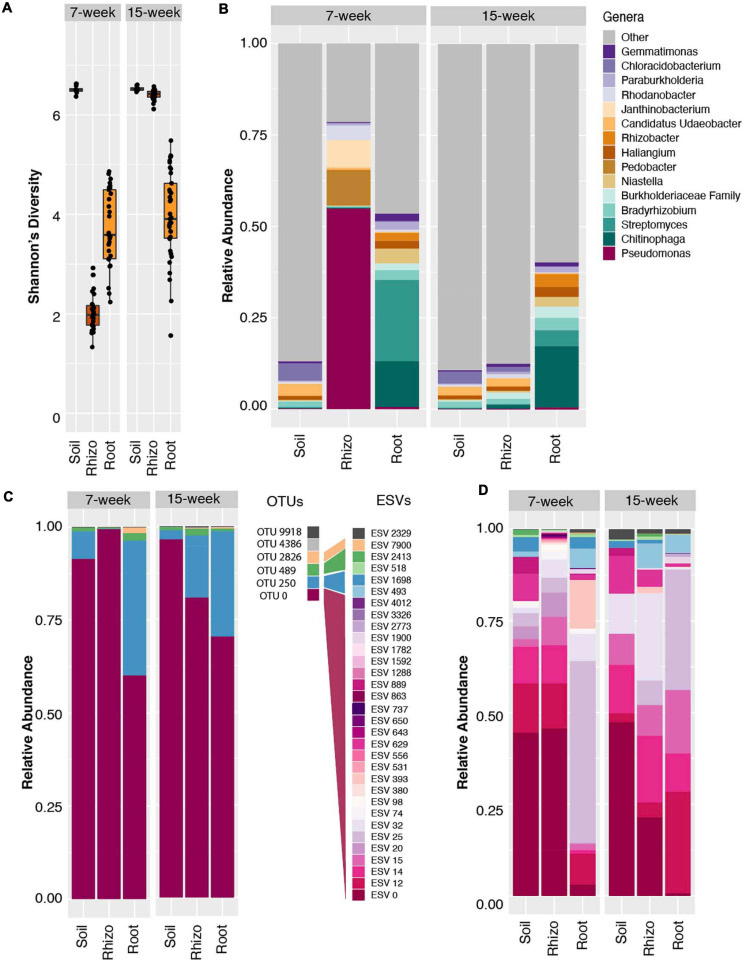
Amplicon data reveals *Pseudomonas* dominance and reduced alpha diversity in 7 week sorghum rhizosphere. **(A)** Boxplots of Shannon’s diversity for soil, rhizosphere and root samples at the 7 week (left panel) and 15 week (right panel) time points using the 97% clustered OTU dataset. **(B)** Relative abundance barplots for the top fifteen most abundant genera in the 97% clustered OTU dataset indicate the *Pseudomonas* genus accounts for ∼60% of the reads within the rhizosphere 7 week samples, but significantly less in other timepoints and sample types, with parallel increases in *Pedobacter* and *Janthinobacter* genera. **(C)** Relative abundance of all six identified *Pseudomonas* OTUs with 97% clustering indicates that the majority of read counts in all sample types map to OTU 0. **(D)** Relative abundance barplots for all *Pseudomonas* ESVs derived from the same 16S amplicon data analyzed using DADA2. Of the resulting 32 *Pseudomonas* ESVs identified in this analysis, 26 (shown in shades of pink and purple) mapped to the dominant OTU 0, as determined by a sequence similarity threshold of 97% in pairwise alignment to all six *Pseudomonas* OTUs. ESVs mapping to three of the other *Pseudomonas* OTUs are indicated with shades of blue, green, and yellow. Note that OTU and ESV numberings are independent of each other.

To assess microbial community diversity across a panel of sorghum genotypes grown under distinct nitrogen fertilization regimes, 16S rRNA amplicon sequencing was performed on field grown sorghum roots, rhizospheres, and associated soils. A total of 10 sorghum cultivars were planted in both high and low nitrogen (N) fields in eastern Nebraska, and collections took place at two time-points [7 and 15 weeks (w) post planting]. We first clustered the amplicon reads into OTUs at 97% sequence similarity, normalized for differences in read depth, and evaluated differences in alpha diversity across sample compartments, nitrogen treatments, and sampling dates. We noted that Shannon’s diversity in the rhizosphere compartment collected at the 7 week (7 w) time point was significantly lower than that observed at 15 weeks (15 w) (ANOVA: *p* < 2e−16, *F* = 5036), with median values less than those observed within the root endosphere (Figure 1A). This corresponded to decreased levels of both Shannon’s evenness and species richness (Supplementary Figures S1A,B). By contrast, rhizospheres at 15 w display levels of alpha diversity were on par with those found in soils, a result typical of field-derived root microbiome studies (Edwards et al., 2015; Naylor et al., 2017). This result demonstrates a shift in overall Shannon’s diversity of the rhizosphere across the time interval, with no corresponding significant changes in the soil or root compartments between the two time points (Figure 1A).

To explore possible taxonomic drivers of the differences in Shannon’s diversity observed within the rhizosphere over the two time points, an analysis of genus-level relative abundance patterns was conducted. Notably, this analysis revealed a significant enrichment in the relative abundance of bacteria from the genus *Pseudomonas* in the 7 w time point rhizosphere samples, which comprised an average of 57% of all microbial reads from these samples (Figure 1B and Supplementary Figure S1C), as compared to other sample types and sampling times. We did not observe a similar relative abundance of *Pseudomonas* in the bulk soil or root endosphere compartments, indicating a potentially plant-driven enrichment that appears specific to the rhizosphere. In this analysis, the majority of reads belonging to the genus *Pseudomonas* mapped to a single OTU (Figure 1C), OTU 0, and these reads comprised on average 48 and 67% of the high and low N rhizosphere reads, respectively (Supplementary Figure S1C). However, by the 15 w time point, this highly abundant OTU represented less than 1% of all reads in the rhizosphere (Supplementary Figure S1C). Interestingly, while this OTU also accounted for 85 and 65% of all *Pseudomonas* reads in the bulk soil and root samples, respectively, at the 7 w time point (Figure 1C), it represented less than 2% of the total reads in these compartments overall at both time points (Figure 1B and Supplementary Figure S1C). Collectively, these results suggest that the reduction in alpha diversity in rhizospheres from the 7 w time point is driven by increases in relative abundance of the bacterial genus *Pseudomonas*, which is represented only at low levels in other sample types and time points.

To investigate possible similarities between the *Pseudomonas* OTUs detected in our study and those previously reported in the rhizosphere compartments of maize, we compared the reference sequence of the single highly abundant *Pseudomonas* OTU in our study to the NCBI NR database. This revealed that the representative sequence of the *Pseudomonas* OTU 0 is identical in terms of nucleotide sequence identity across the V4 16S rRNA region to a wide spectrum of known *Pseudomonas* species, including *P. fluorescens*, *P. brassicacearum*, *P. arsenicoxydans*, and *P. putida*, among others. Interestingly, this OTU is 100% identical to one predominant OTU in a study of maize rhizosphere communities (Walters et al., 2018). Additionally, we noted that the first 90 bases of this OTU are 100% identical to the seventh most abundant 16S rRNA gene tag sequence present in the Earth Microbiome Project (tags in the EMP are 90 bp in total length). This OTU is often observed within plant rhizospheres and animal derived samples across diverse habitats (Thompson et al., 2017). Together, these results suggest that the enriched OTU 0 in this study potentially represents a large group of phylogenetically similar and potentially cosmopolitan bacteria and highlights the need for profiling methodologies capable of providing additional phylogenetic resolution.

Conventional clustering methods in which OTUs are formed through clustering at a 97% nucleotide sequence identity threshold can lead to underestimates of microbial diversity and mask important ecological patterns (García-García et al., 2019); it can be challenging to determine from such analyses if abundance of a given OTU is driven by a single clonal population, or by a diverse collection of related strains or species. To determine if the *Pseudomonas* population enriched within the 7 w rhizospheres is made up of a single or many distinct lineages, we used DADA2 to obtain exact sequence variant (ESV)-level resolution. DADA2 revealed a larger number of ESVs (*n* = 32) that belong to the *Pseudomonas* genus in the dataset as compared to the number of OTUs found in the 97% clustered results (*n* = 6) (Figures 1C,D). By mapping each of the DADA2-derived ESV sequences to the OTUs derived from 97% clustering, we determined that 26 of the 32 ESVs are encompassed within the single dominant 97% identity OTU 0 described above (Figure 1D). Individually, these 26 ESVs represented between 1 and 40% of the overall rhizosphere community at the 7 w time point. Comparisons to *Pseudomonas* ESVs representation in the surrounding soil show marked similarity, despite the large difference in total *Pseudomonas* relative abundance (54% in the rhizosphere vs. 1% in the soil). This suggests that perhaps a shared trait within the core genome of this lineage, rather than strain or species-specific differences in the auxiliary genome (Tettelin et al., 2005), is responsible for its time-dependent success within the rhizosphere. These results differ from those of Karasov et al. (2018) who used a 99% sequence similarity OTU clustering threshold and found that a single *Pseudomonas* OTU dominated the phyllosphere of *Arabidopsis thaliana*. This OTU was classified as *P. viridiflava*, part of the *P. syringae* complex which contains many well characterized leaf pathogens (Hirano and Upper, 2000). It is plausible that this previously characterized low diversity bloom in *Arabidopsis* may represent a classic case of plant-pathogen dynamics; this study highlights potential differences in *Pseudomonas* colonization dynamics that may depend on plant sample type and *Pseudomonas* phylotype. This result also differs from recent findings that highlight strong shifts in the abundance of sub-lineages in response to environmental factors (García-García et al., 2019), which provide evidence that microdiversity may allow stability and survival of the parent lineage across fluctuations in environmental conditions or niches.

**FIGURE 2:**
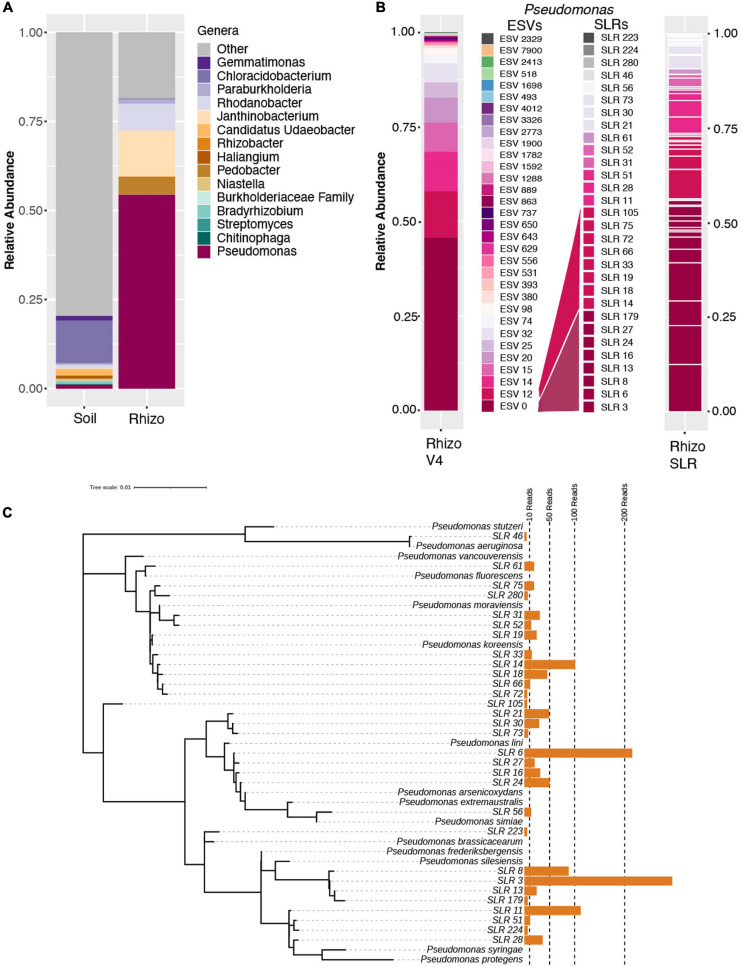
Synthetic long-read amplicon data reveal diversity within the *Pseudomonas* population. **(A)** Relative abundance barplots of the same fifteen genera identified as most abundant in the OTU dataset (Figure 1A) based on single-molecule long-read sequencing data of select 7-week rhizosphere and soil samples. **(B)** Relative abundance barplots of all ESVs (at left, as presented in Figure 1D) and synthetic long reads (SLRs, at right) belonging to the *Pseudomonas* genus. The mapping of SLRs to specific ESVs, as determined by a sequence similarity threshold of 97% in pairwise alignment over the V4 region, is indicated by the color of individual SLRs and their corresponding ESV map. Four SLRs with no identical match to any of the ESVs over the V4 region are indicated with shades of gray. **(C)** Tree of all 32 SLRs belonging to the *Pseudomonas* genus and the taxonomy of their best BLAST hits (considering only SLRs with at least 5 reads across the full dataset after normalization to 1500 reads per sample). Aligned by Muscle and ML Tree constructed in MEGA, visualized in iTOL. SLR 46 (top) is only found in the soil sample and is an outlier at the base of the tree (best match to *Pseudomonas aeruginosa*).

While ESV technology improved the granularity of our clustering results, taxonomic resolution remained in part limited by the short length of the amplified V4 region of the 16S rRNA used in library preparation. To improve taxonomic resolution of the observed *Pseudomonas* expansion, we employed a synthetic long-read (SLR) sequencing technology (LOOP Genomics, San Jose, CA, United States) on a subset rhizosphere and soil samples from the 7 w time point. Amplification using this method yielded a total of 22,208 and 6,019 molecule reads from the rhizosphere and soil, respectively. After read depth normalization, *Pseudomonas* molecules in the rhizospheres were observed to comprise approximately half (57.8%) of the total read counts (Figure 2A), consistent with both the OTU-level and ESV-level analyses of the 16S rRNA V4 amplicon dataset. Additionally, mean values of Shannon’s Diversity were significantly lower in the rhizosphere than in the soil (rhizosphere = 4.85, soil = 6.95, *p* < 0.001), and the relative abundances of the three other most abundant genera in the rhizosphere (*Pedobacter*, *Janthinobacter*, and *Rhodanobacter*) showed similar patterns in both the single-molecule long-read and the V4 datasets (Figures 1B, 2A). Among all of the long-reads belonging to *Pseudomonas*, 390 synthetic long reads (SLRs) were observed more than once in the dataset, and in accordance with the results from our V4 datasets, these *Pseudomonas* molecules comprised less than 2% of total reads in the soil (Figure 2A). The majority of these 390 *Pseudomonas* SLRs were singletons (*n* = 320). Still, considering only SLRs that had at least five reads across the soil and rhizosphere samples still resulted in 30 distinct *Pseudomonas* SLRs that encompassed 75% of all *Pseudomonas* reads. While this number of phylotypes is similar in terms of *Pseudomonas* diversity as that observed in the DADA2 results (Figure 2B), this is likely due to relatively lower read-depth of the SLR dataset compared to that of the ESV dataset. In fact, over the V4 region, many of these SLRs are 100% identical to one another, and the majority are more than 99% similar, which would prevent even ESV level analysis from differentiating them. As a result, these full length SLRs enabled greater differentiation of distinct *Pseudomonas* lineages within the bloom (Figure 2C). Collectively, these analyses highlight the potential advantage of combining ESV-based 16S rRNA amplicon analysis strategies with new single-molecule long-read technologies to help differentiate phylogenetic patterns obscured by traditional OTU-based approaches.

Among the most abundant SLRs (Figure 2C) are phylotypes that have 100% sequence identity matches to known plant-associated *Pseudomonas* species, including *P. silesiensis, P. lini, P. moraviensis*, and *P. frederiksbergensis* (Figure 2C). *Pseudomonas silesiensis* sp. nov. strain A3^T^ was recently discovered as a new species isolated from soil in a biological wastewater treatment plant used by a pesticide packaging company (Kaminski et al., 2018). *Pseudomonas lini* sp. CFBP 5737 was discovered in rhizosphere associated samples (Delorme et al., 2002), and it was recently suggested that this lineage may help to support growth of Lodgepole pine in nitrogen-limited environments (Padda et al., 2019). *Pseudomonas frederiksbergensis* sp. OS261 is a known plant growth promoting bacteria that enhances cold tolerance in tomato (Subramanian et al., 2016) and salt tolerance in pepper (Chatterjee et al., 2017). *Pseudomonas moraviensis* subsp. stanleyae was recently found to colonize the endospheres of coniferous trees and is potentially involved in growth promotion through bioremediation of toxic elements in the soils (Staicu et al., 2015). While these data demonstrate that full length 16S rRNA sequencing better identifies the range of taxonomic diversity in plant microbiomes, which can be useful for subsequent isolation, characterization and mapping efforts, it unfortunately does not often allow for species level identification or inference about putative function. Within many bacterial genera, and *Pseudomonas* in particular, species-level identification and outcomes for host fitness cannot accurately be made even with full length 16S rRNA due to both the taxonomic breadth of the genus and also the strong divergence in functional capacity among even strains with perfect identity across their 16S rRNA sequence similarity (Melnyk et al., 2019).

To further explore the hypothesis that multiple *Pseudomonas* lineages were responsible for the bloom and to determine their taxonomic identities, we performed shotgun metagenomic sequencing on select samples from the 7 w time point (4 from the rhizosphere, 1 from the soil, and 1 from the root endosphere). Multiple strategies for assembly and binning failed to produce any high-quality *Pseudomonas* metagenome assembled genomes, likely due to the highly redundant and abundant sequences of closely related *Pseudomonas* species making full genome resolution challenging. As an alternative to binning, we selected 15 fully sequenced *Pseudomonas* isolates in the Integrated Microbial Genomes (IMG) database based on similarity to the 10 most abundant full-length *Pseudomonas* 16S rRNA sequences in our single-molecule data and mapped the QC-filtered metagenomic reads to these genomes. This analysis revealed that the rhizosphere samples exhibited high levels of mapping to multiple *Pseudomonas* species, including *P. frederiksbergensis* LMG 19851 (IMG database ID: 2636416079), *P. arsenicoxydans* CECT 7543 (IMG database ID: 2636416065, and a relative of *P. lini*) and *Pseudomonas* sp. OV341 (IMG database ID: 2757320528) (Supplementary Figure S2). These results support our finding that the rhizosphere bloom comprises multiple closely related *Pseudomonas* phylotypes and suggests that such a strategy will allow for further resolution of subpopulations as additional *Pseudomonas* isolates are fully sequenced and added to the IMG database.

While the *Pseudomonas* genus has been extensively studied in the context of plant pathogenicity (Höfte and De Vos, 2006), it is now clear that this complex lineage also contains strains with beneficial properties. Multiple *Pseudomonas* species have been identified in disease-suppressive soils with communities of microbes that protect crops from pathogens (Weller et al., 2002). *Pseudomonas* have also recently been shown to support plant growth under a variety of abiotic stresses including drought and salinity (Islam et al., 2014; Egamberdieva et al., 2015; Chandra et al., 2018; Chu et al., 2019). In the study presented here, the diversity of *Pseudomonas* lineages found in the sorghum rhizosphere, as well as the greater abundance of the genera *Pseudomonas* in low nitrogen conditions (Supplementary Figure S1C), point to the possibility that some of these *Pseudomonas* lineages may provide benefits to their host. Interestingly, in addition to the enrichment of the genus *Pseudomonas*, we also observed other taxonomic groups that showed similar though less pronounced enrichment within the 7 w rhizosphere, including the genera *Pedobacter*, *Janthinobacterium*, and *Rhodanobacter* (Figure 1B). Interestingly, Walters et al. (2018) also observed a co-occurrence of *Pedobacter* with *Pseudomonas*. This result suggests that the observed bloom, while dominated by *Pseudomonas*, may involve a collection of diverse microbes with correlated abundance and growth patterns, and future work will help to explore the nature of this relationship.

**FIGURE 3:**
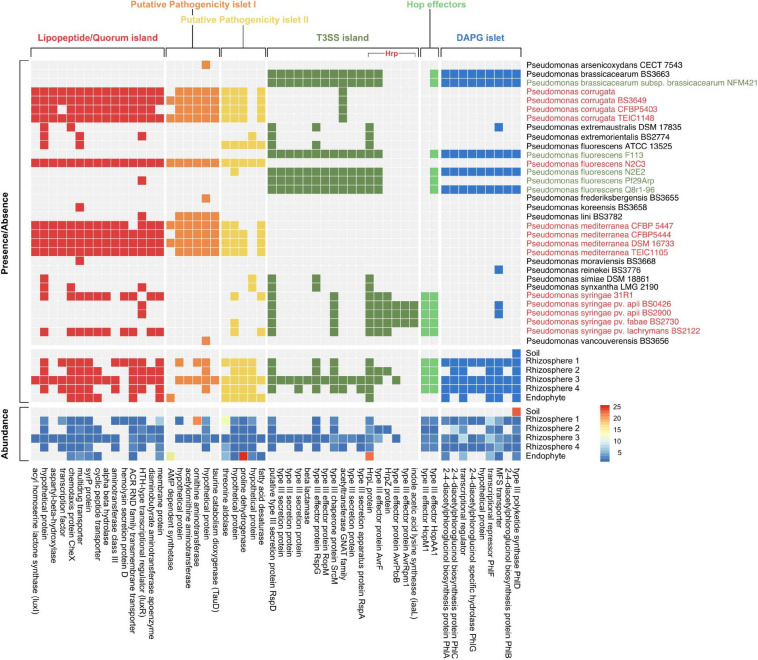
Mapping of metagenome data to *Pseudomonas* gene islands confirms presence of putative pathogenic and commensal genes. Presence/absence of orthologs in isolate genomes from taxa with 16S rRNA sequences closely matched to most abundant SLRs from this study (top panel). *Pseudomonas* isolates identified in green represent isolates with known commensal relationships with plants whereas isolates in red represent known pathogenic isolates. The bottom two panels show both presence/absence and abundance of orthologs in the sorghum soil, rhizosphere, and endosphere metagenomic reads. Proteins associated with each gene in the island are labeled on the bottom of the heatmap.

To characterize whether the relationship between sorghum and *Pseudomonas* at 7 w had the potential to be beneficial or detrimental to plant growth, we identified multiple genomic islands associated with either pathogenic or commensal *Pseudomonas* lifestyles (Melnyk et al., 2019) among a dataset of 140 *Pseudomonas* isolate genomes from the IMG database and our metagenomic sequences (Figure 3). These include three putatively pathogenic loci identified as lipopeptide/quorum-sensing (LPQ) and pathogenicity islets I and II, and three putatively commensal loci identified as type III secretion system (T3SS), Hop effectors, and diacetylphloroglucinol (DAPG) biosynthesis. Given that T3SSs have been associated with both beneficial and pathogenic interactions (Xin et al., 2018), we expanded our analysis of the Hrp family of T3SSs to include both beneficial *Pseudomonas*-associated genes (known as the Rop system) and the virulence genes associated with another member of the *P. fluorescens* lineage, *P. syringae*. We found that while a large proportion of Hrp T3SSs are conserved across both putatively pathogenic and commensal strains, commensal taxa seem to lack a number of genes for the effector (HopM1 and iaaL) and avirulence (Avr) molecules and their associated regulatory molecules, HrpL and HrpZ (Figure 3 and Supplementary Figure S3) that are required for successful infection (Alfano et al., 2000). Additionally, of the five *P. syringae* genomes included in our dataset, two lacked at least some of the effector and avirulence molecules associated with pathogenicity, but instead had a number of genes from the LPQ island which were absent from the other *P. syringae* strains. Specifically, these and other related *Pseudomonas* strains had a gene encoding syringomycin biosynthesis enzyme (*syrP*), which has been identified to contribute to pathogenicity in some *P. syringae* strains (Zhang et al., 1997; Scholz-Schroeder et al., 2001).

Our metagenomes lacked most of these *P. syringae*-associated pathogenic effector/avirulence genes, which is not unexpected given that *P. syringae* is most commonly observed in the aboveground plant phyllosphere. Additionally, T3SSs act physically on the host to translocate effector proteins into plant cells, which may explain why these genes were not present in our soil metagenome. Despite the absence of pathogenic T3SSs in most of our metagenomes, genes from the LPQ island and putative pathogenicity islets I/II were present (Figure 3). The precise function of the genes within both putative pathogenicity islets are largely unknown (Melnyk et al., 2019). However, the presence of the genes consistently predicted to play a role in pathogenesis (e.g., *luxI*/*luxR* and *syrP*) suggests that at least some of *Pseudomonas* strains within the sorghum rhizosphere and endosphere have the potential to form pathogenic relationships with their plant host. Still, the high abundance of DAPG genes (relative to LPQ-associated genes) in our rhizosphere metagenomes supports the idea that plants might select for beneficial *Pseudomonas* in this niche. The DAPG cluster is responsible for the production of many antibiotic compounds associated with biocontrol of phytopathogens, the elicitation of induced systemic resistance in plant hosts, and the modulation of plant hormones as an auxin-mimetic compound (Almario et al., 2017). Together, these results suggest that in addition to selection for commensal taxa by the host, microbe-microbe interactions between commensal and pathogenic strains in the rhizosphere may also influence strain-level composition.

At present, the cause of these recently reported *Pseudomonas* blooms remains unclear. The developmental transition to flowering has been suggested to be a major driver of plant microbiome development (Edwards et al., 2018; Zhalnina et al., 2018). However, in the present study this transition does not appear to correlate with the appearance or disappearance of the *Pseudomonas* bloom. At the 7 w time point, all 10 varietals were in the vegetative state and the bloom was evident in all rhizosphere samples; at the 15 w time point, one half of the varietals had transitioned to flowering, half were still in the vegetative state, and no *Pseudomonas* bloom was observable in any sample (Table 1). We hypothesize that the *Pseudomonas* observed here, and to a lesser extent the other enriched genera, are opportunistic colonizers, existing as copiotrophs that thrive in the developing rhizosphere community following a seasonal or development-gated signaling of exuded metabolites. Indeed, it is known that *Pseudomonas* as a genus is quite specialized in their usage of plant exudates, as they lack phosphofructokinase, the key enzyme in the Embden–Meyerhof–Parnas (EMP) pathway, aka glycolysis, prioritizing sugar processing through the Entner-Doudoroff (ED) pathway (Entner and Doudoroff, 1952; Wang et al., 1959; Vicente and Cánovas, 1973). Recent ^13^C labeling experiments in *P. putida* and *P. protegens* confirmed that the first step in glucose catabolism in *Pseudomonas* sp. is to oxidize glucose to gluconate and 2-ketogluconate through three simultaneous pathways that converge on 6-phosphogluconate feeding the ED pathway (del Castillo et al., 2007; Wilkes et al., 2019). Given this evidence of specialized metabolism in *Pseudomonas*, and evidence that the bloom observed in this study comprises a collection of *Pseudomonas* sublineages, we hypothesized that sorghum plants might positively select for these microbes by producing specific exudate compounds.

**TABLE 1:** *Sorghum bicolor* hybrid and inbred lines and developmental stage at dates of harvest.

**Variety**	**Treatment (High vs. Low N)**	**Days to flowering**	**Developmental stage at 7 w (50 days post planting)**	**Developmental stage at 15 w (104 days post planting)**
PI 297130	High and Low	151	Vegetative	Vegetative
CU-C225	High and Low	151	Vegetative	Vegetative
PI 505735	High N	83	Vegetative	Flowering
PI 505735	Low N	103	Vegetative	Vegetative
PI 506030	High and Low	151	Vegetative	Vegetative
PI 510757	High and Low	151	Vegetative	Vegetative
PI 642998	High N	70	Vegetative	Flowering
PI 642998	Low N	80	Vegetative	Flowering
PI 655972	High N	71	Vegetative	Flowering
PI 655972	Low N	95	Vegetative	Flowering
CU-C053*	High N	83	Vegetative	Flowering
CU-C053*	Low N	95	Vegetative	Flowering
CU-C056*	High N	85	Vegetative	Flowering
CU-C056*	Low N	101	Vegetative	Flowering–just barely
CU-C126*	High N	93	Vegetative	Flowering
CU-C126*	Low N	127	Vegetative	Vegetative

Ten diverse Sorghum bicolor lines were grown in two fields in Mead, NE in the summer of 2015. Hybrid and inbred lines were in the vegetative developmental stage at 7 w when the Pseudomonas bloom was observed and 6/10 of the varietals were still vegetative at 15 w.

*Hybrid lines produced at Clemson University.

**FIGURE 4:**
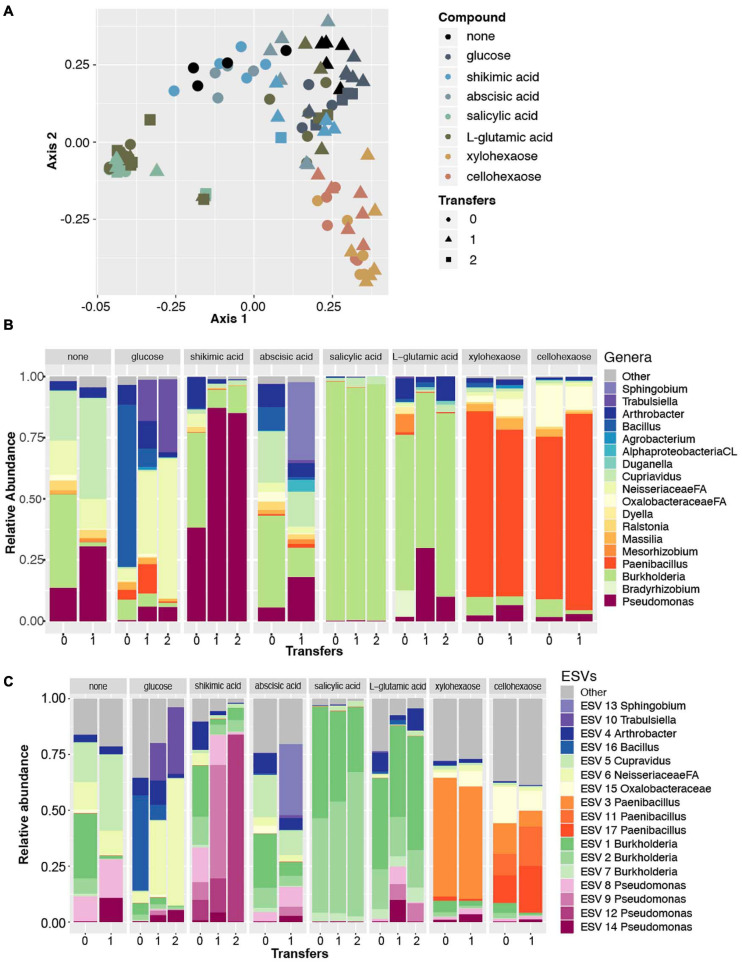
Amplicon data reveals *Pseudomonas* enriched in minimal media with shikimic acid. **(A)** Principal coordinate analysis of all amplicon data across eight enrichment treatments (as indicated by color) and three different numbers of experimental transfers (as indicated by shape). **(B)** Relative abundance of the top eighteen most abundant genera across the dataset for each enrichment compound (facets) and for each distinct number of transfers/subcultures (*x*-axis). Colors for three genera that were also identified as abundant in previous experiments (*Pseudomonas*, *Bradyrhizobium*, *Burkholderia*) are plotted with the same colors as previously; the remaining genera are plotted with new colors. **(C)** Relative abundance plots of individual ESVs within this dataset. Individual ESVs are colored with the same colors used to plot their respective genera in the previous panel. In three cases where multiple ESVs mapped to an individual genus (*Pseudomonas*, *Burkholderia*, and *Paenibacillus*), shades of the previously used color are used to indicate distinct ESVs.

To explore the hypothesis that plant produced carbon compounds may trigger the *Pseudomonas* expansion, we conducted a microbial enrichment experiment using field soil from the original site and a selection of root exudate compounds and plant polysaccharides. In total, seven compounds were selected based on reported involvement in rhizosphere microbiome dynamics [salicylic acid (Lebeis et al., 2015), shikimic acid (Zhalnina et al., 2018)], reported presence in exudates [abscisic acid (Yang et al., 2002), glutamic acid (Forde and Lea, 2007)], and components of plant cell walls (glucose, xylohexaose, cellohexaose) (Scheller and Ulvskov, 2010). Each compound was dissolved in a minimal, defined growth medium (Bruce et al., 1999), and a no carbon source control was included to determine the microbes that are enriched on the base minimal media alone. A soil extract prepared as previously described (Yan et al., 2015) was used as the microbial source material and was added to the sterile minimal media containing each of the seven root exudate compounds and was allowed to grow in the dark at 30°C with 150 rpm shaking until visually turbid (24–48 h). Once turbid, each solution was subcultured to a fresh solution to further enrich with less of the original starting soil extract present. We conducted 16S rRNA sequencing and DADA2-based ESV analysis of these enriched samples and discovered with PCoA analysis that samples clustered very tightly by the compound used in enrichment, shifting slightly with each subculture to new media (Figure 4A).

We found that the only compound that significantly selected for the *Pseudomonas* genus was shikimic acid (Figure 4B). Likewise, an indicator species analysis found that for the shikimic acid compound, only *Pseudomonas* was significantly enriched (no other microbial taxa were enriched, *p* > 0.01). Salicylic acid enriched for the genus *Burkholderia* and the other carbon compounds significantly enriched for a range of other microbial genera (Table 2). Interestingly, when examined at the ESV level, the dominantly enriched ESVs shifted at each transfer of shikimic acid; the initial enrichment had approximately seven co-dominant ESVs, the first transfer had approximately four, and by the second transfer, there was a single ESV (ESV12) that dominated the phylogenetic space (Figure 4C).

**TABLE 2:** Indicator species analysis of microbial genera enriched from field soil with specific carbon treatments in minimal media.

**Genera**	***P*-value**	**Treatment**	**Indicator value**	**Phylum**	**Class**
***Burkholderia***	**0.00010526**	**Abscisic acid**	**0.48334329**	Proteobacteria	Betaproteobacteria
***Rhodococcus***	**0.00084211**	**Abscisic acid**	**0.31150442**	Actinobacteria	Actinobacteria
*Streptomyces*	0.00363158	Abscisic acid	0.27379913	Actinobacteria	Actinobacteria
*Kitasatospora*	0.00394737	Abscisic acid	0.25733691	Actinobacteria	Actinobacteria
*Phenylobacterium*	0.006	Abscisic acid	0.29848905	Proteobacteria	Alphaproteobacteria
*Sphingobium*	0.00721053	Abscisic acid	0.49850285	Proteobacteria	Alphaproteobacteria
*Ramlibacter*	0.00736842	Abscisic acid	0.225	Proteobacteria	Betaproteobacteria
*Novosphingobium*	0.00915789	abscisic acid	0.23226905	Proteobacteria	Alphaproteobacteria
***Paenibacillus***	**5.26E−05**	**Cellohexaose**	**0.67676132**	Firmicutes	Bacilli
***Paenibacillus***	**5.26E−05**	**Cellohexaose**	**0.44552851**	Firmicutes	Bacilli
***Paenibacillus***	**0.00010526**	**Cellohexaose**	**0.63191459**	Firmicutes	Bacilli
***Oxalobacteraceae***	**0.00015789**	**Cellohexaose**	**0.58703868**	Proteobacteria	Betaproteobacteria
***Chitinophaga***	**5.26E−05**	**Glucose**	**0.50604049**	Bacteroidetes	[Saprospirae]
***Stenotrophomonas***	**0.00015789**	**Glucose**	**0.47259238**	Proteobacteria	Gammaproteobacteria
***Paucimonas***	**0.00015789**	**Glucose**	**0.38461538**	Proteobacteria	Betaproteobacteria
***Neisseriaceae***	**0.00026316**	**Glucose**	**0.50906402**	Proteobacteria	Betaproteobacteria
***Bacillus***	**0.00057895**	**glucose**	**0.60755374**	Firmicutes	Bacilli
*Lysobacter*	0.00173684	Glucose	0.29282675	Proteobacteria	Gammaproteobacteria
*PaenibacillaceaeFA*	0.00484211	Glucose	0.26632726	Firmicutes	Bacilli
*Clostridium*	0.00489474	Glucose	0.27451	Firmicutes	Clostridia
***Achromobacter***	**5.26E−05**	**none**	**0.45869095**	Proteobacteria	Betaproteobacteria
***Pimelobacter***	**5.26E−05**	**None**	**0.44444444**	Actinobacteria	Actinobacteria
***Cupriavidus***	**0.00015789**	**None**	**0.55549722**	Proteobacteria	Betaproteobacteria
***Ralstonia***	**0.00036842**	**None**	**0.37328085**	Proteobacteria	Betaproteobacteria
***Methylobacteriaceae***	**0.00047368**	**None**	**0.33333333**	Proteobacteria	Alphaproteobacteria
***Rhodococcus***	**0.00073684**	**None**	**0.33382394**	Actinobacteria	Actinobacteria
***Nocardioidaceae***	**0.00073684**	**None**	**0.30780142**	Actinobacteria	Actinobacteria
*Variovorax*	0.00131579	None	0.34618769	Proteobacteria	Betaproteobacteria
*Labrys*	0.00163158	None	0.31316527	Proteobacteria	Alphaproteobacteria
iii115OR	0.00594737	None	0.22222222	Acidobacteria	Acidobacteria-6
*Vogesella*	0.00626316	None	0.28461467	Proteobacteria	Betaproteobacteria
*RhizobialesOR*	0.00678947	None	0.22222222	Proteobacteria	Alphaproteobacteria
*Methylobacterium*	0.00721053	None	0.22222222	Proteobacteria	Alphaproteobacteria
***Burkholderia***	**5.26E−05**	**Salicylic acid**	**0.40340536**	Proteobacteria	Betaproteobacteria
*Burkholderia*	0.00384211	Salicylic acid	0.30229657	Proteobacteria	Betaproteobacteria
***Pseudomonas***	**5.26E−05**	**Shikimic acid**	**0.5215635**	Proteobacteria	Gammaproteobacteria
***Cohnella***	**5.26E−05**	**Xylohexaose**	**0.86026341**	Firmicutes	Bacilli
***Paenibacillus***	**5.26E−05**	**Xylohexaose**	**0.54333565**	Firmicutes	Bacilli
***Massilia***	**0.00084211**	**Xylohexaose**	**0.40493563**	Proteobacteria	Betaproteobacteria
*Ensifer*	0.00321053	Xylohexaose	0.3556536	Proteobacteria	Alphaproteobacteria
*Agrobacterium*	0.00368421	Xylohexaose	0.27272727	Proteobacteria	Alphaproteobacteria

*Notably, the only carbon compound that enriched for Pseudomonas was shikimic acid and the only microbial genus that shikimic acid significantly enriched for was Pseudomonas. Bolded lines = p-value < 0.001.*

While it is clear from these results that microbes can be enriched by various nutrient sources, it is unclear what causes the *Pseudomonas* growth in the field. Shikimic acid is a precursor in aromatic acid biosynthesis; roots with reduced amounts of this compound were found to be associated with the higher biomass soghum lines under the full N field (Sheflin et al., 2019). However, in that study, metabolites were only measured in the roots, and it’s unclear to what extent that content influenced the exudate profiles. Exudation of shikimic acid from plant roots has been documented to shift across plant development (Chaparro et al., 2013; Zhalnina et al., 2018). Shikimic acid and its precursor, 3-dehydroshikimic acid, was previously found to peak in *Avena barbata* root exudates at weeks 6–9 (vegetative stage) of development (Zhalnina et al., 2018), which is comparable to the stage of growth of our field-grown sorghum plants at 7 w (Table 1).

Additionally, evidence from other systems has shown that plants are capable of interfering with bacterial quorum sensing dynamics (Teplitski et al., 2000; Gao et al., 2003; Mathesius et al., 2003). *Pseudomonas*, like other gram-negative bacteria, are known to rely on acyl-homoserine lactones (AHL) as a primary quorum sensing mechanism (Venturi, 2006), and several studies have found that plants are capable of producing AHL mimics (Teplitski et al., 2000; Mathesius et al., 2003; Degrassi et al., 2007; Veliz-Vallejos et al., 2020). The precise source, structure and biological significance of these mimics remains unclear, but in light of the bloom observed here presents an interesting thread for future investigation.

Taken together, this suggests that the *Pseudomonas* bloom at the 7 w time point may have been due to a combination of environmental conditions, plant development/root exudates, and the genomic ability of a subset of the genus *Pseudomonas* to respond to specific plant produced compounds. Further work including comparative genomics across the *Pseudomonas* genus is essential to tease apart the factors that govern its place in the complex and dynamic nature of the plant rhizosphere.

## Conclusion

The rhizosphere space is a dynamic environment known to be influenced by root exudation. While the diversity of the rhizosphere microbial community is typically lower than that of the surrounding soil and higher than that of the root, there are a small number of reported exceptions (Ofek et al., 2014; Walters et al., 2018) where microbes from the genus *Pseudomonas* dominate the rhizosphere and drastically reduce rhizosphere diversity. Here, we demonstrate such an exception in a 16S rRNA field study of the sorghum root in high and low nitrogen fields near Mead, NE. We discovered a highly abundant *Pseudomonas* population that dominated the early season rhizosphere, was not abundant in the corresponding roots and soils, was independent of sorghum genotype and was present in both high and low nitrogen fields. Rather than this being clonal expansion of a single isolate, long-read 16S sequencing data and shotgun metagenomic data confirm that this population encompasses multiple *Pseudomonas* phylotypes. Using the same field soil brought back to the laboratory and resuspended in water, we were able to enrich for the genus *Pseudomonas* using minimal media and shikimic acid, a precursor in aromatic acid biosynthesis. While the cause of the *Pseudomonas* bloom remains unknown, our results suggest the possibility that plants may select for rhizosphere microbes by exuding specific carbon compounds.

## Materials and Methods

### Field Description

This study was conducted at Eastern Nebraska Research and Extension Center (ENREC) located near Mead, Nebraska in the United States during the summer of 2015. The study was a pilot for a large-scale study to examine the effects of nitrogen limitation on sorghum growth and associated microbial communities. The low N field (41.163166, −96.424108) had not had nitrogen applied for more than 20 years and was in an oat and sorghum rotation with oat forage removed each year. The full N field (41.156414, −96.408031) had eighty pounds per acre of N in the form of anhydrous ammonia applied early in the spring and was in a soybean and sorghum rotation. Both fields were planted on June 2, 2015.

### Plant Germplasm Selection

Ten diverse genotypes of sorghum, representing a range of racial, geographic, and breeding status characteristics, were selected for testing (Table 1). All lines represent bioenergy types and possible candidates for biofuels production. Entries are identified by plant introduction numbers of the U.S. National Plant Germplasm System or hybrid numbers from Clemson University (for more information or germplasm, please contact Dr. Stephen Kresovich at Clemson University).

### Field Sample Collection and Processing

Soil, rhizosphere, and root samples were collected from low and high nitrogen fields on July 22 (7 weeks) and September 15, 2015 (15 weeks). Two plants per genotype were excavated from each of two replicate plots. Soil was removed from the excavated roots and collected. A variety of roots including crown, seminal, and primary roots were excised and had one phosphate buffer wash (6.33 g/L NaH_2_PO_4_, 8.5 g/L Na_2_HPO_4_ anhydrous, 200 μl/L Silwet L-77) to collect rhizosphere samples (McPherson et al., 2018). Harvested roots were then separated for downstream analyses, including microbiome and metabolomics analyses (McPherson et al., 2018).

### Laboratory Preparation of Roots, Soil, and Rhizosphere

Roots were surface sterilized (5.25% sodium hypochlorite + 0.01% Tween 20, 30 s), followed by an ethanol rinse (70% ETOH, 30 s) and three sterile ultrapure water rinses, then stored at −80°C prior to DNA extraction. The rhizosphere samples were filtered through a sterile 100 μm mesh filter (Fisher Scientific, United States), pelleted (3,000 × g, 10 min), and resuspended in 1.5 ml of phosphate buffer (6.33 g/L NaH2PO4, 8.5 g/L Na2HPO4 anhydrous), transferred to a sterile 2 ml microfuge tube, then pelleted (full speed, 5 min), the supernatant removed and the rhizosphere soil pellet was stored at −20°C until DNA extraction. Soil was sieved twice (US Standard Sieve #4, 4750 micron, followed by Sieve #8, 2360 micron) to remove debris and roots then stored at −20°C for DNA extraction.

### DNA Extraction of Soil, Rhizosphere, and Root Samples

DNA was extracted from soil and rhizosphere samples using PowerSoil-318 htp 96 Well Soil DNA Isolation Kit (MoBio, Carlsbad, CA, United States) following the manufacturer’s protocol. DNA was extracted from roots with PowerPlant Pro-htp 96 Well DNA Isolation Kit (MoBio, Carlsbad, CA, United States) following the manufacturer’s protocol. The DNA was quantified with the Quantifluor dsDNA reagent (Promega, Great Lakes, United States) following the manufacturer’s protocol.

### Enrichment of Microbial Taxa With Carbon Compounds

For enrichment experiments, 5 mM of each compound (L-glutamic acid, glucose, cellohexaose, xylohexaose, shikimic acid, salicylic acid, abscisic acid) was dissolved in RCH2 Minimal Fresh Water medium (Bruce et al., 1999) with 0.6 M PIPES buffer (sesquisodium salt, Fisher BP304-500), adjusting the pH to 7.2 and filter sterilizing. A no-carbon source control was included to determine the microbes that are enriched on the minimal media. Fresh soil was collected from the low N field site on July 29, 2019 and stored at 4°C in 50 ml tubes for ∼3 months, then left at room temperature for 1 week, allowing gas exchange prior to soil extraction. The soil extraction protocol was based on Yan et al. (2015); in brief, 20 g of fresh soil and 190 ml of sterile milliQ water were placed in a glass beaker with a stir bar at room temperature and mixed for 15 min and filtered through a sterile coffee filter. This solution was called the 10-1 dilution. Five milliliters of the 10-1 dilution was transferred to a 50 ml tube with 45 ml of sterile milliQ water, and the tube was inverted to mix to make the 10-2 dilution. Initial enrichment cultures were established with 3 ml of media (with each dissolved carbon compound) in 15 ml conical tubes (BD Biosciences, San Jose, CA, United States) with 300 μl of soil extract and grown in the dark at 30°C with 150 rpm shaking until visually turbid. For glucose and L-glutamic acid, visual turbidity was at 24 h. For cellohexaose, xylohexaose, shikimic acid, salicylic acid, and abscisic acid, turbidity was reached at 48 h. For the no-compound control, samples were harvested after 48 h for initial enrichment and 5 days for first transfer (no visual turbidity at 5 days). For transfers, a 1:100 dilution was made (transferring 30 μl of enrichment culture to 3 ml of new growth media) once culture was turbid with microbial growth.

### Amplification and Illumina Sequencing of 16S Tag Sequences

DNA was quantified and amplified in 96 well plates with single indexed primers targeting the V4 region of the bacterial 16S rRNA gene (Walters et al., 2016, 2018). Chloroplast and mitochondrial Peptide Nucleic Acid (PNA) blockers were used to prevent chloroplast and mitochondrial amplification in all samples (Lundberg et al., 2013). Amplified samples were multiplexed at 184 samples per PE 2 × 300 Illumina MiSeq sequencing run. For the enrichment experiments, DNA was quantified and amplified in 96 well plates with dual-indexed primers targeting the V4/V5 region of the bacterial 16S rRNA gene (Thompson et al., 2017).

### iTagger Amplicon Analysis

Data from the sequencer was demultiplexed and processed through bbduk for end trimming, quality filtering and masking^1^. High quality reads were processed by iTagger version 2.2 (Tremblay et al., 2015). Version 2.2 processes sequencing amplicon data by iteratively clustering reads at 99, 98, and 97% identity using the USEARCH software suite (Edgar, 2010). Samples with less than 500 total reads were discarded from downstream analysis, and the resulting OTUs were classified using the RDP classifier (Wang et al., 2007) trained with a custom version of the Silva database (release v132, Quast et al., 2013) dereplicated at 99% identity and trimmed to the V4 region of the 16S rRNA. ESVs matching mitochondria, chloroplasts, and those which were not classifiable below the Kingdom level, were removed prior to downstream statistical analyses. The source code for iTagger is available on Bitbucket: http://bitbucket.org/berkeleylab/jgi_itagger. For normalization purposes and to remove low abundance OTUs, we kept OTUs in the downstream analysis that had at least two reads in at least five samples and normalized the remaining dataset by randomly subsampling each sample to a consistent read depth of 20,000 reads per sample. The analysis of Shannon’s Diversity in Figure 1A was conducted using the *diversity* function in the R package *vegan* (Oksanen et al., 2019*)*, and the subsequent test of significant differences by month within the rhizosphere samples was performed using an ANOVA as implemented by the *aov* function in the base R stats package.

### DADA2 Amplicon Analysis

For DADA2 analysis, raw data was processed in QIIME2 (Caporaso et al., 2010) training the error rate model with 330,000 reads (corresponding to ∼100 million nucleotides). Resulting ESVs were classified using the RDP classifier (Wang et al., 2007) trained on a custom version of the Silva database (release v132, Quast et al., 2013) dereplicated at 99% identity and trimmed to the V4 region of the 16S rRNA. ESVs matching mitochondria, chloroplasts, and those which were not classifiable below the Kingdom level, were removed prior to downstream statistical analyses. To account for differences in read depth across sample types, the resulting data set was filtered using the same parameters as for the iTagger analysis: all ESVs not observed at least 5 times in at least 2 samples were discarded, and samples were normalized by rarefaction to 20,000 reads per sample.

### 16S rRNA Single-Molecule Long-Read Processing and Analysis

Libraries for sequencing were made using the LoopSeq^TM^ 16S and 18S Microbiome 24-plex Kit (Loop Genomics, San Jose, CA), following the manufacturer’s instructions. In brief, barcoding adapters were assigned to each of seven genomic DNA samples (10 ng, including two soil, two rhizosphere), where each 16S and 18S rRNA gene molecule received its unique barcode. The samples were then pooled into one tube, and the barcoded molecules were amplified using PCR. After random distribution of the barcode within each 16S/18S molecule, the DNA was enzymatically fragmented and ligated with adapters for sequencing on Illumina platform. Raw data for seven samples from the LOOP single-molecule platform was concatenated into a single FASTA sequence, for a total of 77,927 sequences. Unique sequences (*n* = 52,817) were obtained using USEARCH (Edgar, 2010) and UNOISE was used to cluster all sequences using a minsize parameter of 1, resulting in 11,035 non-chimeric SLRs. Taxonomic assignment was performed using the RDP classifier (Wang et al., 2007) trained on a custom version of the Silva database (Quast et al., 2013) dereplicated at 99% identity and trimmed to the V4 region of the 16S rRNA.

### Shotgun Metagenomic Sequencing

For rhizosphere samples, 100 ng of DNA was sheared to 300 bp using the Covaris LE220 and size selected using SPRI beads (Beckman Coulter). The fragments were treated with end-repair, A-tailing, and ligation of Illumina compatible adapters (IDT, Inc.) using the KAPA-Illumina library creation kit (KAPA biosystems). The prepared libraries were quantified using KAPA Biosystem’s next-generation sequencing library qPCR kit and run on a Roche LightCycler 480 real-time PCR instrument. The quantified libraries were then prepared for sequencing on the Illumina HiSeq sequencing platform utilizing a TruSeq paired-end cluster kit, v4. Sequencing of the flow cell was performed on the Illumina HiSeq2500 sequencer using HiSeq TruSeq SBS sequencing kits, v4, following a 2 × 151 indexed run recipe.

For root and soil samples, plate-based DNA library preparation for Illumina sequencing was performed on the PerkinElmer Sciclone NGS robotic liquid handling system using Kapa Biosystems library preparation kit. 200 ng of sample DNA was sheared to 600 bp using a Covaris LE220 focused-ultrasonicator. The sheared DNA fragments were size selected by double-SPRI and then the selected fragments were end-repaired, A-tailed, and ligated with Illumina compatible sequencing adaptors from IDT containing a unique molecular index barcode for each sample library. The prepared libraries were quantified using KAPA Biosystem’s next-generation sequencing library qPCR kit and run on a Roche LightCycler 480 real-time PCR instrument. The quantified libraries were then prepared for sequencing on the Illumina HiSeq sequencing platform utilizing a TruSeq paired-end cluster kit, v4. Sequencing of the flow cell was performed on the Illumina HiSeq2500 sequencer using HiSeq TruSeq SBS sequencing kits, v4, following a 2 × 151 indexed run recipe.

### Genome Wide Mapping of Field Metagenomic Reads to Isolate Genomes

The 10 most abundant 16S sequences identified through the long-read 16S data were compared against isolate genomes in the IMG database^2^. The genomes with above 99% similarity to the most abundant 16S were selected as references (Table 3). The QC-filtered metagenomic reads were then mapped to these complete genomes using BBMAP (Bushnell, 2014) to generate statistics on nucleotide identity and genome coverage.

**TABLE 3:** Reference isolate genomes of 15 fully sequenced *Pseudomonas* isolates most similar to the 10 most abundant *Pseudomonas* sequences in our single-molecule long-read 16S data.

**IMG_ID**	**Species**	**Genome size**	**Gene count**	**Scaffold count**	**16S rRNA count**	**Matched to**	**Matched ratio (matched sequences/total sequences)**
2645727914	*Pseudomonas fluorescens* C3	6699120	6,224	60	1	SLR3	1,491/1,497
2636415920	*Pseudomonas fluorescens* H24	6899099	6,409	168	1	SLR3	1,487/1,497
2744054431	*Pseudomonas* sp. 655	7168167	6,436	51	1	SLR3	1,487/1,497
2757320528	*Pseudomonas* sp. OV341	6938938	6,444	59	1	SLR3	1,486/1,497
2718218023	*Pseudomonas frederiksbergensis* AS1	6208705	5,909	2	7	SLR3	1,485/1,497
2636416079	*Pseudomonas frederiksbergensis* LMG 19851	6394336	6,014	2	8	SLR3	1,483/1,497
2636416065	*Pseudomonas arsenicoxydans* CECT 7543	6502878	6,237	1	5	SLR6	1,493/1,494
2663762789	*Pseudomonas prosekii* LMG 26867	6091955	5,643	1	4	SLR6	1,492/1,494
2823489066	*Pseudomonas frederiksbergensis* KNU-15	6595804	6,161	1	6	SLR11	1,497/1,497
2821271981	*Pseudomonas umsogensis* KD5_MF50	6501538	6,065	10	6	SLR11	1,497/1,497
2852657418	*Pseudomonas* sp. JAI115	6472974	5,937	34	1	SLR14	1,494/1,495
2721755610	*Pseudomonas koreensis* CRS05-R5	5991225	5,579	1	6	SLR14	1,493/1,495
2579779090	*Pseudomonas* sp. RIT288	6273290	5,616	44	1	SLR14	1,493/1,495
2784132063	*Pseudomonas* sp. 424	6262788	5,866	1	6	SLR14	1,493/1,495
2639762619	*Pseudomonas jessenii* LMG 21605	6588452	6,218	2	9	SLR14	1,489/1,495

### Ortholog Detection in Metagenomic Reads

Orthologs were predicted using PyParanoid v 0.4 (Melnyk et al., 2019). A dataset containing 140 high quality *Pseudomonas* isolate genomes from the IMG database (Study ID: Gs0114533) was used to build the reference pangenome. In brief, DIAMOND (Buchfink et al., 2015) and InParanoid (Sonnhammer and Östlund, 2015) were used to generate pairwise similarity scores and identify pairwise orthology relationships. Gene families were then identified using Markov clustering (mcl) and aligned using MUSCLE (Edgar, 2004). Alignments were used to build hidden Markov models (HMMs) using HMMER3 (Eddy, 2011) and these HMMs were used to propagate ortholog annotations of our metagenomes. A presence/absence map of a subset of putatively pathogenic and commensal gene islands identified in Melnyk et al. (2019) was generated using the NMF package in R v 4.0.2 (Gaujoux and Seoighe, 2010).

### Statistical Analysis

Multivariate analysis for date and N treatment factors was completed for both rhizosphere bacterial composition and root metabolite profiles using the adonis2 function in the vegan package (Oksanen et al., 2019) for R statistics software (R). Pairwise distances for these comparisons were calculated using the Bray Curtis ordination method (Beals, 1984). A two-way ANOVA univariate test was used to determine differential relative abundance of a single OTU in rhizosphere soil by date, treatment or the interaction in Prism 7 (GraphPad, La Jolla, CA, United States). Correlation analyses utilized the cor function in R and with the non-parametric Spearman’s rank option. The *corrplot* package (Wei and Simko, 2017) for R was used to visualize correlation analyses as heatmaps. Scripts for all microbiome related statistical analysis are available at the github repository https://github.com/dawn-chiniquy/Chiniquy-2020.

## Data Availability Statement

The datasets presented in this study can be found in online repositories. The names of the repository/repositories and accession number(s) can be found below: https://www.ncbi.nlm.nih.gov/, SRP130758; https://www.ncbi.nlm.nih.gov/, SRP130755; https://www.ncbi.nlm.nih.gov/, SRP130757; https://www.ncbi.nlm.nih.gov/, SRP130767; https://www.ncbi.nlm.nih.gov/, SRP130775; https://www.ncbi.nlm.nih.gov/, SRP135653; https://www.ncbi.nlm.nih.gov/, SRP165130; and https://www.ncbi.nlm.nih.gov/, PRJNA692505.

## Author Contributions

DC, AD, ST, and DS designed the experiments. DC, EM, AP, AS, DS, ST, and JP collected the data. DC, JZ, XL, and EB analyzed the data. DC wrote the manuscript. KH, EB, AS, AD, DS, and ST critically reviewed the manuscript. All authors contributed to the article and approved the submitted version.

## Conflict of Interest

The authors declare that the research was conducted in the absence of any commercial or financial relationships that could be construed as a potential conflict of interest.

## Footnotes

1 https://jgi.doe.gov/data-and-tools/bbtools/bb-tools-user-guide/bbduk-guide/

2 https://img.jgi.doe.gov/cgi-bin/m/main.cgi

## Supplementary Material

Supplementary Figure 1 Amplicon data reveals components of Shannon’s diversity and *Pseudomonas* expansion across nitrogen treatments in the sorghum rhizosphere. **(A)** Boxplots of Shannon’s evenness for soil, rhizosphere and root samples at the 7 week (left panel) and 15 week (right panel) time points using the 97% clustered OTU dataset. **(B)** Boxplots of species richness for soil, rhizosphere and root samples at the 7 week (left panel) and 15 week (right panel) time points using the 97% clustered OTU dataset. **(C)** Relative abundance barplots for the top fifteen most abundant genera in the 97% clustered OTU dataset as plotted for individual rhizosphere samples within the 7 week time point (left two panels) and 15 week time point (right two panels) for each of the two nitrogen treatments (individual panels). These data demonstrate a large increase in *Rhodanobacter* in high nitrogen samples that is not seen in the low nitrogen samples (ANOVA, *p* < 0.001, *F* = 45.93). Additionally, these data demonstrate that while *Janthinobacterium* abundance remains relatively equivalent between treatment groups in the rhizosphere 7 w time point (ANOVA, *p* = 0.62, *F* = 0.26), the genus *Pedobacter* is higher in the high nitrogen compared to low nitrogen (ANOVA, *p* < 0.01, *F* = 10.31) and the genus *Pseudomonas* is higher in the low nitrogen than high nitrogen (ANOVA, *p* < 0.001, *F* = 16.49).

Supplementary Figure 2 Genome wide alignment of assembled shotgun metagenomic reads with 15 closest isolate genomes based on long read 16S data. Plots of metagenome data for individual field samples selected for metagenomic sequencing: **(A)** rhizosphere 7 week time point from low N field. **(B–D)** three replicates of rhizosphere 7 week time point from high N field. **(E)**, soil 7 week time point from low N field, **(F)**, root 7 week time point from low N field. For each panel, 15 fully sequenced *Pseudomonas* isolates were selected from the IMG database based on similarity to the 10 most abundant full-length *Pseudomonas* sequences in our single-molecule data and subsequently we mapped the QC-filtered raw metagenomic reads to these genomes. Plots represent the proportion of mapped reads (*y*-axis) as a function of mapped percent identity (*x*-axis) for 15 selected genomes. The majority of rhizosphere samples exhibit high levels of mapping to multiple *Pseudomonas* species, including *P. frederiksbergensis* LMG 19851 (IMG database ID: 2636416079), *P. arsenicoxydans* CECT 7543 (IMG database ID: 2636416065, and a relative of *P. lini*) and *Pseudomonas* sp. OV341 (IMG database ID: 2757320528).

Supplementary Figure 3 Presence/absence of Hrp-family T3SS orthologs in *Pseudomonas* isolate genomes and sorghum metagenomes. Isolates identified in green represent isolates with known commensal relationships with plants whereas isolates in red represent known pathogenic isolates. Proteins associated with each gene in the island are labeled on the bottom of the heatmap.
