# Supplementary material for: Microbial Community Field Surveys Reveal Abundant Pseudomonas Population in Sorghum Rhizosphere Composed of Many Closely Related Phylotypes
Source: Front Microbiol. 2021 Mar 9;12:598180. doi: 10.3389/fmicb.2021.598180 (PMC7985074; doi:10.3389/fmicb.2021.598180)
Supplement: Supplementary Figure 1 — Amplicon data reveals components of Shannon’s diversity and Pseudomonas expansion across nitrogen treatments in the sorghum rhizosphere. (A) Boxplots of Shannon’s evenness for soil, rhizosphere and root samples at the 7 week (left panel) and 15 week (right panel) time points using the 97% clustered OTU dataset. (B) Boxplots of species richness for soil, rhizosphere and root samples at the 7 week (left panel) and 15 week (right panel) time points using the 97% clustered OTU dataset. (C) Relative abundance barplots for the top fifteen most abundant genera in the 97% clustered OTU dataset as plotted for individual rhizosphere samples within the 7 week time point (left two panels) and 15 week time point (right two panels) for each of the two nitrogen treatments (individual panels). These data demonstrate a large increase in Rhodanobacter in high nitrogen samples that is not seen in the low nitrogen samples (ANOVA, p < 0.001, F = 45.93). Additionally, these data demonstrate that while Janthinobacterium abundance remains relatively equivalent between treatment groups in the rhizosphere 7 w time point (ANOVA, p = 0.62, F = 0.26), the genus Pedobacter is higher in the high nitrogen compared to low nitrogen (ANOVA, p < 0.01, F = 10.31) and the genus Pseudomonas is higher in the low nitrogen than high nitrogen (ANOVA, p < 0.001, F = 16.49). [file Data_Sheet_1.PDF]

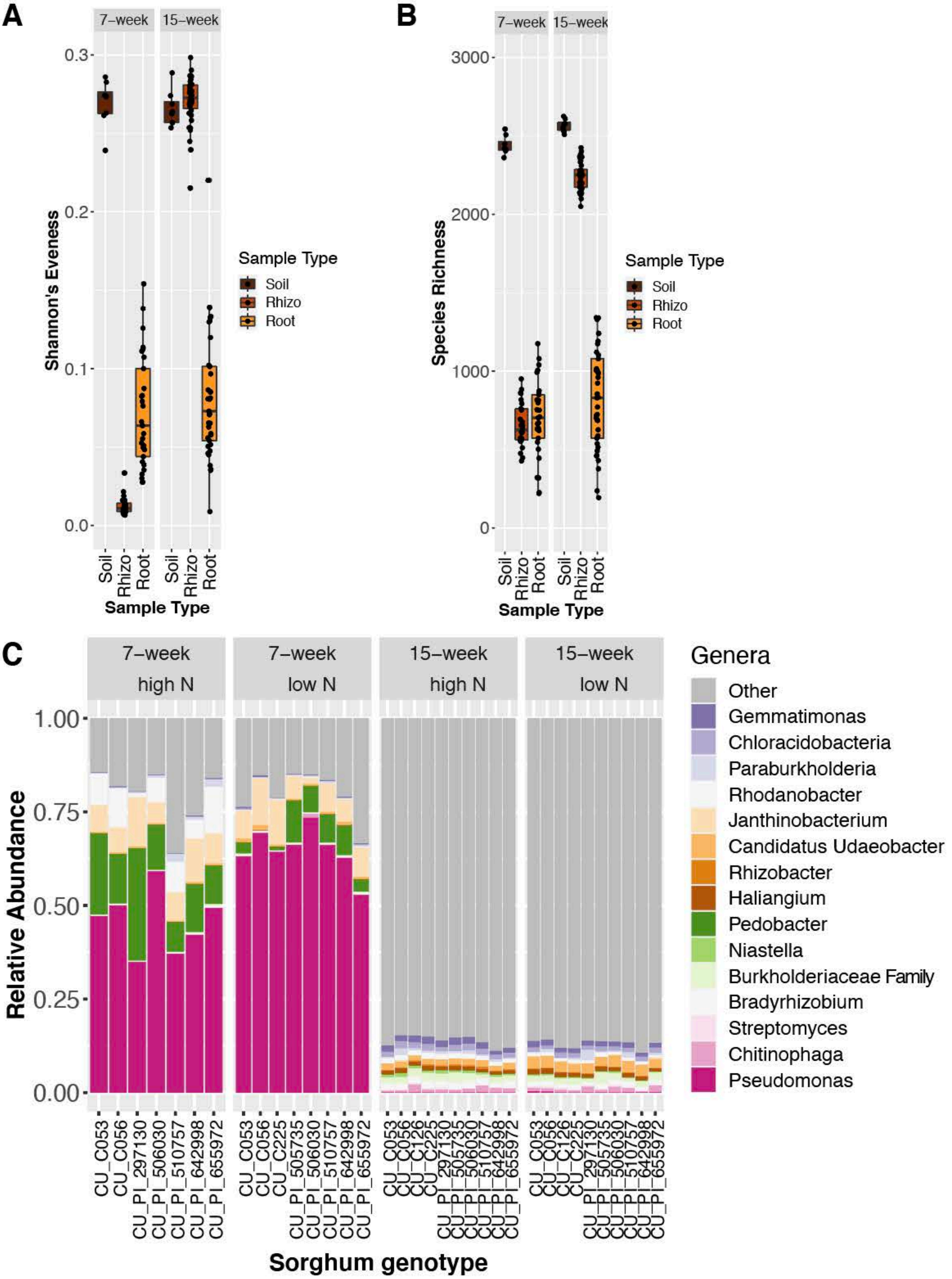


**Supplemental Figure S1. Amplicon data reveals components of Shannon's diversity and Pseudomonas expansion across nitrogen treatments in the sorghum rhizosphere. A,** Boxplots of Shannon's evenness for soil, rhizosphere and root samples at the 7-week (left panel) and 15-week (right panel) time points using the 97% clustered OTU dataset. **B,** Boxplots of species richness for soil, rhizosphere and root samples at the 7-week (left panel) and 15-week (right panel) time points using the 97% clustered OTU dataset. **C,** Relative abundance barplots for the top fifteen most abundant genera in the 97% clustered OTU dataset as plotted for individual rhizosphere samples within the 7-week time point (left two panels) and 15-week time point (right two panels) for each of the two nitrogen treatments (individual panels). These data demonstrate a large increase in *Rhodanobacter* in high nitrogen samples that is not seen in the low nitrogen samples (ANOVA, $p$ <0.001, $F$=45.93). Additionally, these data demonstrate that while *Janthinobacterium* abundance remains relatively equivalent between treatment groups in the rhizosphere 7w time point (ANOVA, $p$ =0.62, $F$=0.26), the genus *Pedobacter* is higher in the high nitrogen compared to low nitrogen (ANOVA, $p$ <0.01, $F$=10.31) and the genus *Pseudomonas* is higher in the low nitrogen than high nitrogen (ANOVA, $p$ < 0.001, $F$=16.49).

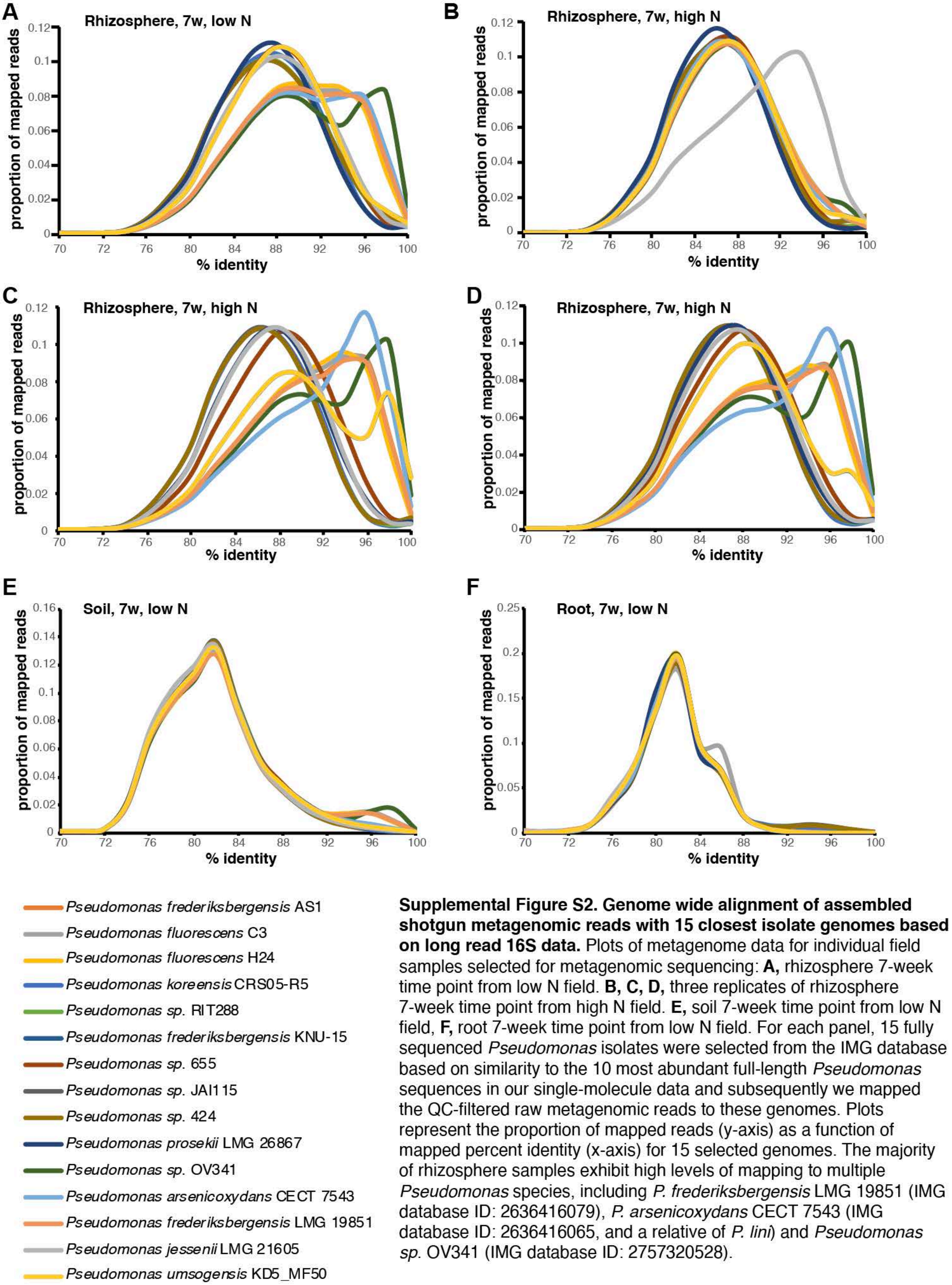


**Supplemental Figure S2. Genome wide alignment of assembled shotgun metagenomic reads with 15 closest isolate genomes based on long read 16S data.** Plots of metagenome data for individual field samples selected for metagenomic sequencing: **A,** rhizosphere 7-week time point from low N field. **B, C, D,** three replicates of rhizosphere 7-week time point from high N field. **E,** soil 7-week time point from low N field, **F,** root 7-week time point from low N field. For each panel, 15 fully sequenced *Pseudomonas* isolates were selected from the IMG database based on similarity to the 10 most abundant full-length *Pseudomonas* sequences in our single-molecule data and subsequently we mapped the QC-filtered raw metagenomic reads to these genomes. Plots represent the proportion of mapped reads (y-axis) as a function of mapped percent identity (x-axis) for 15 selected genomes. The majority of rhizosphere samples exhibit high levels of mapping to multiple *Pseudomonas* species, including *P. frederiksbergensis* LMG 19851 (IMG database ID: 2636416079), *P. arsenicoxydans* CECT 7543 (IMG database ID: 2636416065, and a relative of *P. lini*) and *Pseudomonas sp.* OV341 (IMG database ID: 2757320528).

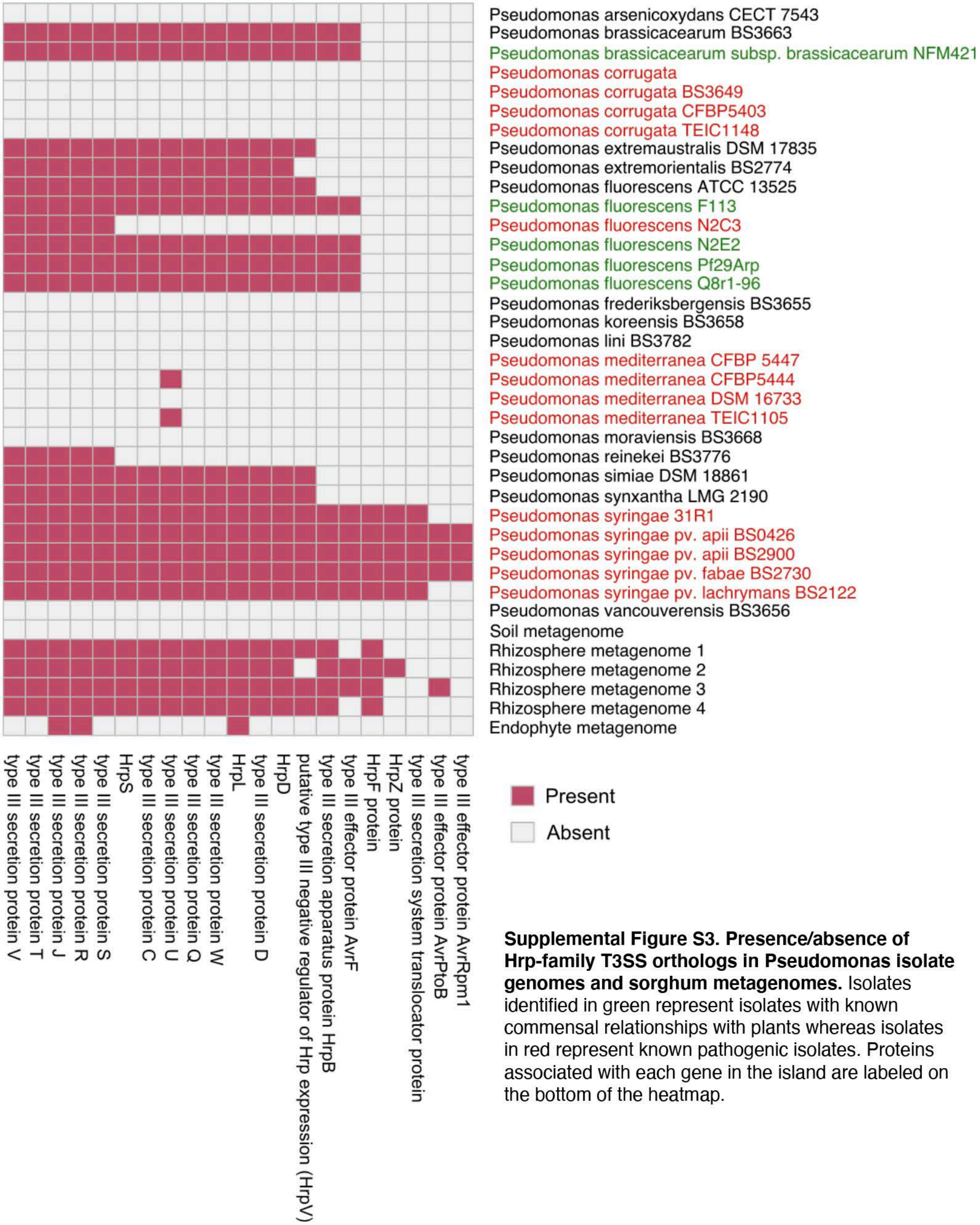


**Supplemental Figure S3. Presence/absence of Hrp-family T3SS orthologs in Pseudomonas isolate genomes and sorghum metagenomes.** Isolates identified in green represent isolates with known commensal relationships with plants whereas isolates in red represent known pathogenic isolates. Proteins associated with each gene in the island are labeled on the bottom of the heatmap.
